# Boerhaave Syndrome, Pneumothorax, and Chylothorax in a Critically Ill Patient with Tuberous Sclerosis Complex

**DOI:** 10.1155/2015/509094

**Published:** 2015-10-15

**Authors:** Mohsin Ijaz, Arsalan Rafiq, Sindhaghatta Venkatram, Gilda Diaz-Fuentes

**Affiliations:** ^1^Division of Critical Care Medicine, Montefiore Medical Center, Albert Einstein College of Medicine, Bronx, NY 10467, USA; ^2^Division of Cardiology, Mount Sinai St. Luke's Hospital, New York, NY 10025, USA; ^3^Division of Pulmonary and Critical Care Medicine, Bronx-Lebanon Hospital Center, Bronx, NY 10457, USA

## Abstract

Tuberous sclerosis complex (TSC) is an autosomal dominant, variably expressed multisystem disease. The predominant pulmonary features of TSC are identical to those of lymphangioleiomyomatosis (LAM). Pneumothorax, multifocal micronodular pneumocyte hyperplasia, and chylothorax are rare complications of TSC. We report a young male with pneumothorax, lung nodules, and chylous effusion who developed empyema thoracis after esophageal rupture. Hospital course was complicated by respiratory failure. Family opted to transfer to hospice care. Chylothorax is a rare complication of TSC with few scattered reports mostly in female patients. Patients with TSC are usually managed by multispecialists and it is important to be aware of the rare pulmonary manifestations of this disease. A male patient with TSC having lung nodules presenting with chylothorax and empyema thoracis from Boerhaave syndrome makes our case unique.

## 1. Introduction

Tuberous sclerosis complex (TSC) is a rare autosomal dominant, multisystem disorder that usually involves brain, heart, skin, eyes, lungs, liver, and kidneys [1]. Genes involved are TSC1 and TSC2 which encode hamartin and tuberin, respectively. Tuberous sclerosis complex was first described in 1862 by Von Recklinghausen [2]. This complex is involved in the inhibition of the mammalian-target-of-Rapamycin (mTOR) pathway which is involved in the control of cell proliferation and growth. Pulmonary manifestations of TSC include pulmonary cysts, multifocal micronodular pneumocytes (MMPH), and lymphangioleiomyomatosis (LAM) [3]. Chylothorax is the accumulation of chyle or lymph from the thoracic duct in the pleural space. This lymphocyte-predominant fluid can accumulate in the pleural space due to leakage from the thoracic duct [4]. Chylothorax can be seen in patients with pulmonary complications of tuberous sclerosis.

## 2. Case Discussion

A 21-year-old male patient was admitted to the intensive care unit with persistent vomiting of four days' duration, associated with progressive dyspnea, dry cough, and loss of appetite. As per the family, there was no history of fever, abdominal pain, dysuria, palpitations, chest pain, or any other associated complaints. There was no history of sick contacts or recent travel and he was up-to-date with immunizations. Medical history was significant for tuberous sclerosis with retinal hamartomas, renal mass, mental retardation, complex partial epilepsy, quadriparesis, severe kyphoscoliosis, iron deficiency anemia, reflux esophagitis with esophageal ulcers, chronic gastritis, and multiple pulmonary nodules.

His home medications included acetaminophen, carbamazepine, ferrous sulphate, folic acid, oral cyanocobalamin, ascorbic acid, multivitamins, omeprazole, and short acting bronchodilators. Patient was born in Dominican Republic and moved to USA at the age of 15; he has been wheelchair-bound since birth. He was born with normal spontaneous vaginal delivery but required oxygen supplementation for one week after birth. He had recurrent seizures since he was an infant. He had 3 siblings; elder brother also suffers from recurrent seizures.

**Figure 1:**
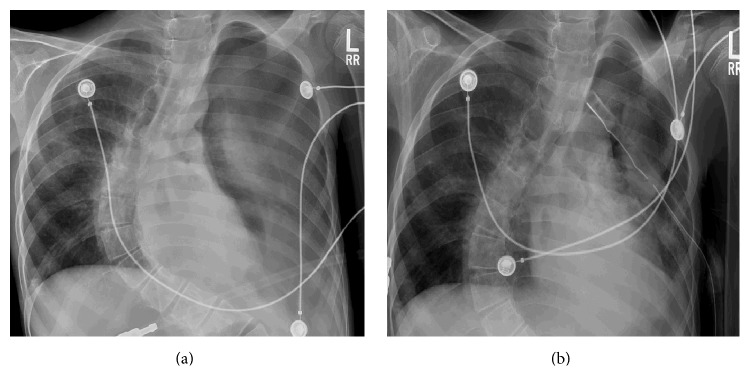
CXR showing (a) left side pneumothorax and pleural effusion. (b) Expansion of the left lung, chest tube, and left sided effusion.

**Figure 2:**
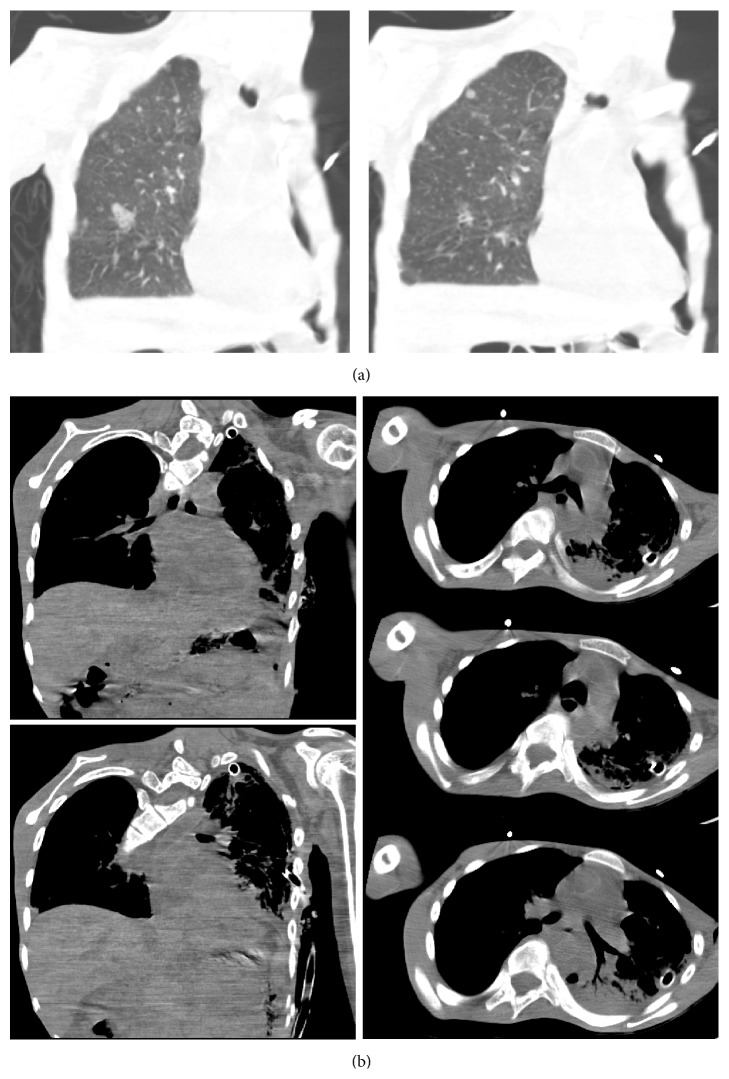
(a) Chest CT different cuts from lung showing right side multiple small nodules. (b) Mediastinal window showing no signs of air in the mediastinum.

On examination, the patient was hypoxic (oxygen saturation of 88% on ambient air which improved to 92% on 2 liters oxygen), tachycardic (heart rate of 120/min), tachypneic (respiratory rate of 30/min), and hypotensive (blood pressure of 80/60 mmHg). Other findings on physical examination included decreased air entry on the left hemithorax, quadriparesis, and slow mentation. Chest X-ray (CXR) revealed large left hydropneumothorax (Figure 1(a)). A chest tube was inserted with 500 mL of purulent, brownish-green fluid drained; followup CXR showed expanded lung with residual pneumothorax (Figure 1(b)). Patient was treated for severe sepsis. Initial pleural fluid was exudative, neutrophilic with negative cytology for malignant cells (Table 1). A chest computerized tomography (CT) showed left pneumothorax with chest tube and multiple small nodules in right lung (Figure 2(a), lung window). There was no evidence of mediastinal air (Figure 2(b)). Subsequent pleural fluid drainage was noted to be milky and confirmed to be chylothorax with triglyceride level of 1041 mg/dL. Subcutaneous octreotide and medium chain triglycerides infusion were started and patient was started on orogastric tube feeding. Cultures from pleural fluid grew heavy growth of* Candida albicans* and Methicillin sensitive* Staphylococcus aureus* confirming the effusion to be empyema thoracis. Fluconazole and nafcillin were initiated and initial antibiotics (vancomycin and cefepime) were discontinued. Pleural fluid drainage remained elevated with daily output of 1.5–2 liters of chylous fluid/day despite octreotide.

**Figure 3:**
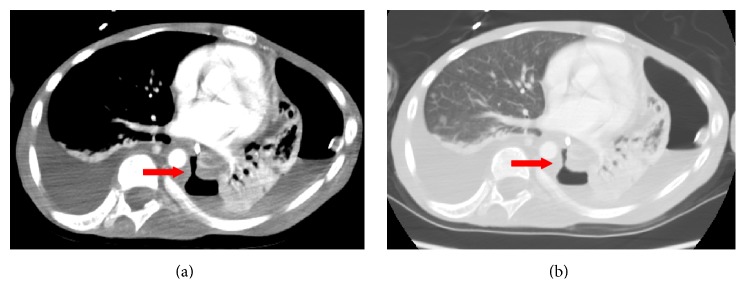
Chest CT with wide window width: (a) mediastinal window, (b) lung window showing esophageal tear and communication with pleura (red arrow).

**Figure 4:**
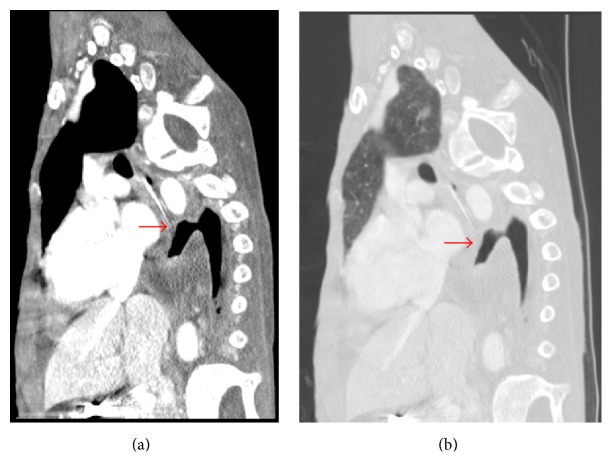
Chest CT: (a) mediastinal window, (b) lung window. Sagittal view showing esophageal tear and communication with pleura (red arrow).

A repeat chest CT scan with contrast 12 days later from the first CT scan revealed increasing left hydropneumothorax. Direct communication between the esophagus and left pleural space was seen, highly suggestive of esophageal tear (Figures 3 and 4). Repeat pleural fluid analysis showed amylase level of 12238 U/L also suggestive of esophageal tear.

Surgical intervention for management of the esophageal tear with possible repair of the thoracic duct was discussed with family who opted for conservative management and hospice care where he later died. We hypothesize that our patient initially presented with pneumothorax and empyema in the background of TSC related lung nodules. Chylothorax was probably related to a combination of lung disease and esophageal tear due to chronic esophagitis and esophageal ulcers.

## 3. Discussion

TSC is characterized by mental retardation, seizures, and cutaneous angiofibromas (Vogt's triad). Diagnosis is made when two major features, or one major and two minor features, are present [1]. Other manifestations include the most common renal angiomyolipomas (80% cases), isolated renal cysts, autosomal dominant polycystic kidney disease, and renal cell carcinoma. Retinal hamartomas and cardiac rhabdomyomas have been described in up to 50% of cases [1].

The pulmonary involvement in TSC is rare, seen in less than 1%, and is secondary to occlusion of the bronchus, vascular, and lymphatics by immature smooth muscle cells [5]. The three main pulmonary lesions found in TSC are LAM, MMPH, and clear cell tumor of the lung, with LAM being the most common. Average age of onset of pulmonary manifestation is 32 to 34 years of age and manifests mainly in women with TSC [6].

Dyspnea with mild exertion, spontaneous pneumothorax, or cough can be the first symptoms of lung involvement in patients with TSC. Progression to pulmonary failure usually does not occur until the third or fourth decade of life with some patients requiring lung transplantation. Clinical, radiological, and pathological manifestations of lung involvement in tuberous sclerosis and LAM are indistinguishable [7]. Radiological features include pneumothoraces, pleural effusion, ground glass opacification, and nodules [5].

Effort related esophageal perforation is usually called Boerhaave syndrome and is associated with high morbidity and mortality. In a study, spontaneous rupture of esophagus due to barotrauma resulting from increase in intraesophageal pressure due to vomiting represented 8 to 56% of all esophageal perforations [8]. The overall reported mortality of esophageal perforations is close to 20%. The intrathoracic rupture of esophagus can involve the overlying pleura due to inflammation and can cause a direct communication between the esophagus and the pleural cavity resulting in pleural effusions [8]. Management options include medical management and endoscopic or surgical repair.

The pleural fluid analysis in our patient also showed evidence of chylothorax, which is an uncommon condition resulting from either traumatic or nontraumatic damage of the thoracic duct. Trauma to the thoracic duct due to penetrating injuries, hyperextension of spine, and complications of surgeries involving the left side of neck, esophagus, and other cardiothoracic surgeries has been reported. These leaks usually heal spontaneously within 10–14 days [4, 9]. Nontraumatic chylothorax has been reported in association with malignancies, particularly lymphomas. Rare causes include subclavian vein thrombosis, filariasis, and thoracic duct tumors. The incidence of chylothorax in patients with LAM is as high as 75% [10]. The mechanism of formation of chylothorax in TSC is not well understood; proposed mechanisms include chyle leak, general oozing from pleural lymphatics or central vessels, and transdiaphragmatic flow of chylous ascites [11].

Pathological lung findings in patients with TSC are similar to LAM and include multiple air-filled small cysts comprised of bundles of smooth muscles with angiomatous new formation. Adenomatoid epithelial proliferation is a characteristic feature. Other findings include MMPH which is considered the second major form of pulmonary involvement in patients with TSC after LAM. MMPH represents benign overexpression of type II pneumocytes along with alveolar septa that reveal fibrous thickening, increase in elastic fibers, and aggregates of alveolar macrophages. MMPH has an unclear prognosis and is very unlikely to have malignant progression, so management is conservative [12].

Management of pulmonary complications of TSC represents a challenge; due to the rarity of the condition, there are no established guidelines. Conservative management of chylothorax is indicated with avoidance of hypoproteinemia and electrolyte imbalance and monitoring for superimposed sepsis [13]. Success rate of conservative management is 16–75% [14]. Other modalities to reduce lymph flow include use of somatostatin or its analog octreotide. Response is assessed by a decrease in the output drain to around half within 48 hours as there are no prospective studies to guide evidence-based management [14]. Surgical interventions are indicated if other measures fail, and they include ligation of the thoracic duct, pleurectomy, pleurodesis, and pleuroperitoneal shunt [15]. Promising is the discovery of upregulation of the mTOR pathway in tumors associated with TSC and the use of mTOR inhibitors like sirolimus or analogs in the management of TSC [16].

Our patient developed sepsis secondary to empyema thoracis. The cause of empyema was initially unrecognized as the initial chest CT scan did not reveal evidence of esophageal rupture, which was found in the subsequent scan. We were not able to find any association between esophageal rupture and empyema thoracis in patients with tuberous sclerosis, as per our search of English literature. Our patient presented with persistent nausea and vomiting, likely leading to esophageal rupture and subsequent development of empyema thoracis causing patient's demise. Unfortunately, family opted for hospice care and no further evaluation or treatment was done.

## 4. Conclusions

Patients with TSC and pulmonary manifestations represent a diagnostic and management challenge with an overall poor prognosis. A male patient with TSC having lung nodules (likely MMPH) presenting with chylothorax and empyema thoracis from Boerhaave syndrome constitutes an extremely rare presentation and has not been reported before. ICU physicians caring for TSC patients should be aware of this presentation and consider a multidisciplinary approach towards early diagnosis and management in order to improve the overall grim prognosis.

## Figures and Tables

**Table 1:** Description of pleural fluid analysis.

Pleural fluid	Day 1	Day 5	Day 20
pH	7.15		
Protein g/dL	3	2.1	
LDH U/L	5656	3628	
Glucose mg/dL	4	247	116
WBC	174000		
RBC	300		
Neutrophils %	96		
Lymphocytes %	4		
Cholesterol mL	—	15	
Triglycerides meq/L	—	1041	11
Amylase U/L	—		12238
Serum			
Protein g/dL	5.1	5.3	
LDH U/L	206		

## Abbreviations

TSC: Tuberous sclerosis complex
mTOR: Mammalian-target-of-Rapamycin
LAM: Lymphangioleiomyomatosis
CT: Computerized tomography
MMPH: Multifocal micronodular pneumocyte hyperplasia.
